# The Quest for Novel Antimicrobial Compounds: Emerging Trends in Research, Development, and Technologies

**DOI:** 10.3390/antibiotics8010008

**Published:** 2019-01-24

**Authors:** Pavan K. Mantravadi, Karunakaran A. Kalesh, Renwick C. J. Dobson, André O. Hudson, Anutthaman Parthasarathy

**Affiliations:** 1 Analytical Services, Abon Pharmaceuticals, Northvale, NJ 07647, USA; pavankumar.mantravadi@gmail.com; 2 Department of Chemistry, Durham University, Durham DH1 3LE, UK; kalesh.karunkaran@durham.ac.uk; 3 Biomolecular Interaction Centre and School of Biological Sciences, University of Canterbury, Private Bag 4800 Christchurch, New Zealand; renwick.dobson@canterbury.ac.nz; 4 Rochester Institute of Technology, Thomas H. Gosnell School of Life Sciences, 85 Lomb Memorial Dr, Rochester, NY 14623, USA

**Keywords:** antibiotics, resistance, antibiotic targets, antimicrobial materials, microbiome

## Abstract

Pathogenic antibiotic resistant bacteria pose one of the most important health challenges of the 21st century. The overuse and abuse of antibiotics coupled with the natural evolutionary processes of bacteria has led to this crisis. Only incremental advances in antibiotic development have occurred over the last 30 years. Novel classes of molecules, such as engineered antibodies, antibiotic enhancers, siderophore conjugates, engineered phages, photo-switchable antibiotics, and genome editing facilitated by the CRISPR/Cas system, are providing new avenues to facilitate the development of antimicrobial therapies. The informatics revolution is transforming research and development efforts to discover novel antibiotics. The explosion of nanotechnology and micro-engineering is driving the invention of antimicrobial materials, enabling the cultivation of “uncultivable” microbes and creating specific and rapid diagnostic technologies. Finally, a revival in the ecological aspects of microbial disease management, the growth of prebiotics, and integrated management based on the “One Health” model, provide additional avenues to manage this health crisis. These, and future scientific and technological developments, must be coupled and aligned with sound policy and public awareness to address the risks posed by rising antibiotic resistance.

## 1. Introduction

Bacteria that are resistant to clinically relevant antibiotics are a major threat to public health worldwide. Factors leading to this crisis, such as the lack of research and development of novel antibiotics, have been examined elsewhere [1,2,3]. The recent global status of antibiotics has been documented in a policy document issued by the Center for Disease Dynamics, Economics, and Policy (CDDEP) [4]. The development of antibiotics was rapid between 1940–1962, when 20 clinically relevant classes were discovered and developed; only two major novel classes have been commercialized since 1962. Large pharmaceutical companies in recent decades have committed scant resources to antibiotics [5], as drugs treating lifestyle diseases yield more sustainable profits. Meanwhile, academic scientists, smaller companies, non-profits, and academic-industry partnerships have brought about significant developments in the fields of antibiotics and antimicrobial strategies. 

For example, the tuberculosis-focused TB Alliance initiated Phase I trials for TBA-7371, which belongs to a new class of compounds called DprE1 inhibitors that block the enzyme decaprenylphosphoryl-β-d-ribose 2′-epimerase (DprE1) in the cell wall biosynthesis of *Mycobacterium tuberculosis*. Only two TB drugs have been approved for clinical use in recent years, namely, Johnson and Johnson’s bedaquiline and Otsuka Pharmaceutical’s delamanid, both for treating multi-drug-resistant tuberculosis. More needs to be done on this front as resistance to known drugs is rising. A new business model for the development of antibiotics that is not dependent on high returns from the large volume product sales has been advocated [6]. This article offers snapshots from the gamut of recent antimicrobial science and technology, including antimicrobial discovery strategies, novel antibiotic types, newer antimicrobial materials, emerging commercial technology platforms, and microbial ecology management, including microbiome interventions. This article is restricted to developments mainly arising in the last decade, and earlier approaches that are being revived due to increasing antibiotic resistance. 

## 2. Novel Targets, Discovery Approaches, and Sources

### 2.1. Novel Antibiotic Targets

Traditionally, antibiotics have targeted mainly bacterial DNA replication, protein synthesis, or peptidoglycan synthesis. Widespread resistance has caused researchers to search for newer targets for combating bacterial infections; an overview is shown in Table 1. The nine nutritionally essential amino acids, namely, leucine, isoleucine, valine, threonine, methionine, tryptophan, phenylalanine, histidine, and lysine are synthesized by bacteria, but not by humans. In addition to protein synthesis, lysine is also involved in bacterial peptidoglycan biosynthesis. The *L*,*L*-diaminopimelate aminotransferase (DapL) pathway for lysine biosynthesis was recently identified as a novel variant occurring in pathogenic genera such as *Chlamydia*, *Leptospira*, and *Treponema* [7]. DapL inhibitors are potential narrow-spectrum antibiotics given the narrow distribution of the enzyme among the bacterial kingdom [8]. Aromatic amino acid biosynthesis also affords multiple antibiotic targets. Four shikimate pathway enzymes are essential for the gastric pathogen, *Helicobacter pylori*; shikimate kinase and type II dehydroquinase are important for both *Mycobacterium tuberculosis* and *Helicobacter pylori*, and inhibitors of these enzymes are under development [9]. The Tuberculosis Structural Genomics Consortium (TBSGC) has facilitated the solving of X-ray diffraction structures of many *Mycobacterium tuberculosis* proteins, including several from the tryptophan biosynthetic pathway, namely, TrpB, TrpC, and TrpE, and inhibitory molecules were discovered [10,11,12]. The shikimate pathway is also essential for parasitic protozoa, such as *Toxoplasma gondii* and *Plasmodium falciparum*, and identifying the relevant genes could spur the production of selective anti-parasitic molecules [13,14]. 

The generation of spontaneous suppressor mutants and a comparison of the genomes of telomycin-sensitive versus resistant cells of *Staphylococcus aureus* and *Bacillus subtilis* revealed mutations in genes encoding for the phospholipid cardiolipin, suggesting cardiolipin biosynthesis as a new antibiotic target [15]. Teixobactin is a new antibiotic that inhibits cell-wall biosynthesis by binding the lipid-pyrophosphate-sugar motif of Lipid II (precursor of peptidoglycan), and of Lipid III of wall teichoic acid in Gram-positive bacteria. Since the structure of Lipid II is not expected to change much via mutations, teixobactin is predicted to be free from bacterial resistance [16]. The compound POL70780 is a *Pseudomonas aeruginosa*-specific inhibitor of LptD, a protein involved in lipopolysaccharide insertion to the outer membrane [17]. Since LptD is well-conserved across bacterial species and the rate of mutations is low, similar compounds could be developed against other pathogens. A recent study identified a tripeptide “staphylopine” in *Staphylococcus aureus* that binds nickel, cobalt, zinc, copper, and iron [18]. The biosynthetic pathway for this peptide was elucidated and several genes in the corresponding gene cluster were conserved in other pathogens, such as *Yersinsia pestis* and *Pseudomonas aeruginosa*, suggesting that molecules interfering with staphylopine biosynthesis could potentially act as broad-spectrum antibiotics. 

Quorum sensing (QS) is a process whereby bacterial signalling molecules are produced and detected in order to coordinate cell-density based behavioral changes [19]. An estimated 80% of all microbial infections are associated with biofilms. Disrupting QS can prevent the formation of biofilms, increasing the importance of quorum sensing inhibitors (QSI). Since QS is not directly involved in cell wall biosynthesis or DNA replication, as anti-virulence factors QSI afford an alternative mechanism compared to traditional antibiotics. The major QS systems are the acyl-homoserine lactone (AHL) of Gram-negative bacteria [19], the auto-inducing peptide (AIP) system of Gram-positive bacteria [20], and the autoinducer-2 (AI-2), which occurs in both groups [21,22]. Inhibitors of the Lux protein family interfere with AHL-based QS and prevent biofilm formation, which enables the antibiotic azithromycin to clear chronic *Pseudomonas* infections in animal models [23]. Substrate analogues and inhibitors of all three QS systems have been reviewed elsewhere [24]. Notably, two proteins in the AI-2 pathway, LuxS and 5’methylthioadenosine nucleosidase (MTAN), are found in bacteria but not in mammals, leaving further scope for using these as targets for new antibiotics [25,26,27,28]. Enzymes, such as acylases, lactonases, and oxidoreductases can degrade or modify AHL signals, a phenomenon termed quorum quenching (QQ) [29]. QQ enzymes are found in bacteria, plants, and mammals. The non-AHL QS systems are not susceptible to QQ, but they have been targeted by antibodies against AIP produced by *Staphylococcus aureus* [30] and AI-2 produced by *Salmonella enterica* serovar Typhimurium [31]. 

Lassomycin is a new peptide active against *Mycobacterium tuberculosis* produced by an *Actinomycete, Lentsea* sp [32]. It inhibits an essential protease ClpP1P2C1 and increases its ATP-ase activity, thereby killing both growing and dormant cells. Traditional antibiotics usually only kill actively growing cells and persistence of dormant cells remains a recurring problem. The acetyldepsipeptide ADEP4 activates the ClpP protease that degrades misfolded proteins with the help of ATP-dependent chaperones [33]. ADEP4 and its analogues, however keep the channel of ClpP open, obviating the need for ATP hydrolysis. Thus, ADEP4 became the first antibiotic to effectively kill persister cells in biofilms. Although resistance to ADEP4 is common, combining it with other antibiotics eradicated biofilms in vitro and in mouse models. Well-known food items, like spices, also contain antibiotic compounds, whose mechanisms of action are being investigated. Ginger contains raffinose, which inhibits biofilms of *Pseudomonas aeruginosa* by decreasing the intracellular levels of cyclic-di-GMP, thus inducing a switch from non-motile (biofilm) to motile (planktonic) cells [34]. Growth of the pathogen is not affected, suggesting that limiting cyclic-di-GMP could be a way to clear biofilm formation. 

### 2.2. Novel Discovery Approaches

#### 2.2.1. Informatics-Based Discovery Approaches 

Newer genomics approaches are beginning to overtake old “Grind and Find” methods of first cultivating microbes and then extracting compounds in the hunt for new antibiotics [35]. A platform that allows the mining of antibiotic biosynthetic gene clusters (BGC) based on similarity to known examples from plants, fungi, and bacteria is the antibiotics and Secondary Metabolite Analysis Shell (antiSMASH) tool [36,37]. Another recent portal mines the genes encoding for the biosynthesis of a particular natural product and uses that knowledge to probe whether or not other strains can make specific important intermediates of the natural product [38]. Alternatively, mining with narrow focus on genes involved in the final few steps of biosynthetic pathways enables the identification and optimization of variants, potentially producing an entire class of analogues using the results of just one study. 

Genomic analysis can also reveal the presence of repressors or activators of biosynthesis genes, paving the way for engineering strains with overproduction capabilities [39]. However, only a fraction of bacterial diversity is routinely cultivated, and therefore, only a small percentage of the chemistries have been sampled. Many existing bioinformatics platforms have a bias towards known molecules, adding to the redundancy of frequent rediscovery. Among *Actinomycetes*, *Streptomyces* is over-represented due to sampling bias arising from different sources [40]. Shotgun sequencing of soil metagenomes remains an unsatisfactory tool for antibiotic discovery, since it remains challenging to sequence deep enough to obtain usable information about BGC [41]. Recently, a barcoding approach using degenerate polymerase chain reaction (PCR) primers to parse mixtures of BGC present in environmental samples via the environmental Surveyor of Natural Product Diversity (eSNaPD) server was formulated [42]. A survey of calcium-dependent antibiotics in diverse soil metagenomes based on eSNAPD analysis showed that more than 70% of BGC were unique to individual metagenomes, and led to the discovery of the malacidins, a novel family of antibiotics, which did not develop resistance under laboratory conditions [43]. 

A comprehensive bio- and chemo-informatics study of all currently known antibiotics was published, wherein 54 distinct molecular targets were identified [15]. The authors created a public web application called “Antibioticome” that defines the chemical origins and families of known natural antibacterials and their targets. An algorithm described as “retrobiosynthetic”, i.e., capable of identifying the chemical building blocks from which each antibiotic originates, was used. Since metadata pertaining to known or suspected mechanisms of action were also incorporated, the method was able to identify candidates with unknown modes of action. Antibiotic resistance often originates from the biosynthetic gene clusters in antibiotic-producers. Therefore, another web application was created to correlate self-resistance with known antibacterial mechanisms allowed for the scanning of BGC for homology to resistance genes and predict antibiotic targets. The application was able to correctly predict that the non-ribosomal peptide telomycin had a potentially unique mechanism of action [15]. The ease and economy of genome sequencing and the development of bioinformatics tools to analyze genomes for antibiotic resistance genes could enable the elucidation of new antibiotic targets. Platforms to detect antibiotic resistance genes in genomes and metagenomes, such as DeepARG and the Resistance Gene Identifier based on the Comprehensive Antibiotic Resistance Database, RGI CARD, have been created [44,45]. 

#### 2.2.2. BGC Activation and Engineering 

Engineering known modular BGCs that assemble specific parts of existing antibiotics to yield novel variants of well-known antimicrobials is an established approach, with the most notable recent example being the production of a novel group of semi-synthetic rifamycins to fight multi-drug resistant *Mycobacterium tuberculosis* [46]. However, the potential for secondary metabolite production is estimated to be much larger than the number of biochemically characterized pathways. The genome sequences of many bacteria contain homologues of known antibiotic biosynthesis genes, such as those encoding polyketide synthases and non-ribosomal peptide synthases. For example, the *Actinomycetes, Streptomyces coelicolor*, and *Sterptomyces avermitilis* harbor, respectively, 20 and 30 operons for secondary metabolites, although only a few are characterized [47,48]. Genome mining enables the identification of silent or cryptic BGC which may not be expressed under standard laboratory cultures. Approaches to activate silent or cryptic BGC responsible for producing novel metabolites have been reviewed recently [49]. 

The major pathway specific approaches are the manipulation of regulatory genes controlling the expression of specific pathways, the replacement of native promoters with constitutive ones, and the use of heterologous expression systems. For example, the silent spectinabilin gene cluster has been refactored by eliminating all the native regulatory control elements and replacing them with a series of constitutive and inducible heterologous promoters, triggering the production of the previously silent end product [50]. Two novel anthraquinone aminoglycosides, Gaudimycins D and E, were discovered by inducing the expression of the *pga* gene cluster in *Streptomyces* sp. PGA64, using a promoter-reporter system which identifies randomly generated mutants, wherein the target BGC has been activated [51]. A genome minimized *Streptomyces* strain has been constructed to facilitate the expression of antibiotic BGC [52]. Methods to express large natural product BGC clusters in *Streptomyces* strains have been recently optimized, and antibiotic production thereby achieved [53,54,55]. 

Pleiotropic approaches alter the expression of multiple genes, inducing global changes in the regulation of specific metabolic pathways. The major pleiotropic strategies are the variation of growth conditions and co-cultivation, epigenetic manipulation, engineering of global regulatory elements, and the modification of the transcription and translation machinery. Varying the growth conditions has been successfully used in many recent examples of antibiotic discovery. An older strategy is upregulating the transcription and translation of cryptic BGC by selecting for mutations in the genes encoding for RNA polymerase (RNAP) or ribosomal proteins. For example, mutations in the ribosomal protein S12 in *Streptomyces lividans* induced the biosynthesis of the antibiotic actinorhodin [56]. A newer approach is the chemical manipulation of the epigenome of fungi with inhibitors of histone deacetylase or DNA methyltransferases in order to trigger the production of novel natural products. When *Penicillium citreonigrum* was treated with the DNA methylase inhibitor 5-azacytidine, novel terpene and polyketide natural products were produced [57]. Using an “old antibiotics for new” approach, adding sub-lethal concentrations of clinical antibiotics was shown to activate otherwise unexpressed BGC for novel antibiotics in strains of *Burkholderia thailandensis* [58,59]. Apart from undefined elicitors that trigger antibiotic production, chemically defined molecules have been used as inducers of antibiotic production [60,61,62]. QS signals were shown to be required to induce the biosynthesis of the antibiotic pyrrolnitrin [63]. Compounds in chemical libraries or screens for blocking defined pathways have also been utilized to elicit antibiotic production, for example by inhibiting fatty acid biosynthesis [61,62]. 

### 2.3. Novel Antimicrobial Sources

Researchers are increasingly finding therapeutics from sources long considered unlikely or exotic. The use of plants used in traditional medicines, marine invertebrates, as well as insects and vertebrate animals on land as potential sources for antimicrobials, is undergoing a revival. Recently, an *Entotheonella* species residing in a marine sponge was found to synthesize more than ten different antibiotic compounds [64]. Due to a long history of co-evolution, the bacterial symbionts of macroflora and fauna could become a source of novel antibiotics [40]. Gut bacteria of the common pest, the cotton leaf-worm, were shown to produce the peptide antibiotic mundticin [65]. Insect gut bacteria might, thus, become a new source of antimicrobial peptides (AMP), with potential applications in food additives and antimicrobial coatings. Amphibians, such as rainforest frogs, resist bacterial and viral infections on their slimy skin and the activities of 14 antiviral peptides from different amphibians were tested against HIV using human T-cell cultures [66]. An AMP was discovered in the blood of the Komodo dragon *Varanus komodoensis*, a large reptile able to effectively resist the infection of open wounds. The natural peptide was taken as a starting point to design a more potent synthetic peptide, also having anti-biofilm activity [67]. *Staphylococcus lugdunensis*, a commensal of the human nasal cavity, produces the antibiotic lugdunin [68]. A new Streptomycete, *Streptomyces formicae*, isolated from the African fungus-growing plant-ant, was shown to produce 16 novel polyketide antibiotics called “formicamycins” [69]. Insect-associated microbes also produce the antibiotics dentigerumycin [70] and napthomycin K [71]. Current literature reports continue to describe endophytic fungal extracts with antimicrobial activity [72,73,74]. Analysis of genomes of endophytic bacteria recently isolated from Jamaican sugarcane using the antiSMASH platform reveals potential candidate BGC for antibiotics [75]. 

Fungi-fungi, bacteria-bacteria, and bacteria-fungi co-cultures produce secondary metabolites not biosynthesized under standard laboratory conditions, and approximately half of the identified metabolites induced during microbial co-cultivation, as opposed to monocultures, showed chemical novelty [76]. Co-cultures are an increasingly important source of new antibiotics [76,77,78]. Cross-species induction has been also achieved by exposing bacteria on the surface of marine algae or invertebrates (epibiotic bacteria) or marine fungi to terrestrial bacteria [79,80,81,82]. Pestalone is a molecule produced by cross-species induction and its total synthesis has been achieved and derivatives have been synthesized [83]. Closthioamide is a novel sulphur-containing antibiotic active against pathogens, including MRSA and vancomycin-resistant enterococci, produced by the anaerobe *Clostridium cellulolyticum* when it is stimulated by sterile-filtered aqueous soil extracts [84]. Interspecies crosstalk via chemical messengers between microbes occupying the same environment may lead to the expression of BGCs not seen under laboratory conditions, even in well-known microorganisms. For example, *Aspergillus nidulans* was shown to produce the compounds F-9775A and F-9775B when co-cultured with soil actinobacteria; these compounds were initially isolated from *Paecilomyces carneus* and were not known *Aspergillus nidulans* metabolites [85]. 

In nature, many growth factors are secreted by members of the microbial community surrounding the organism one is seeking to isolate. Since the biochemistry of these factors is largely unknown, it is unsurprising that traditional cultivation media mostly fail to produce colonies of previously uncultured bacteria. Several recent platforms use microfluidic chambers to separate individual cells derived from environmental samples. The bacteria are kept separate by means of capillary forces, while the growth factors diffuse freely. Thus, “uncultivable” bacteria can grow unhindered in the chambers since their natural environment is at least partly simulated. Microfluidic approaches succeeded in cultivating a previously uncultivated strain, *Eleftheria terrae*, which produces the novel antibiotic teixobactin [16]. A high-throughput version of a diffusion chamber device, the I-chip platform, enables 15–40% of cells to form colonies on agar after repeated inoculation into fresh chips [86]. A large proportion of the colonies grown were novel and did not grow by traditional agar-plate methods. Culturing even a fraction of the previously uncultured majority of bacteria could substantially expand the chemical diversity of antibiotics. 

Venoms (protein and peptide mixtures) from different animals, such as snakes, bees, scorpions, spiders, and poison frogs, also have antibiotic activity. Melittin is a 26-amino acid bee venom peptide that inhibits several pathogens, like *Borrelia burgdorferi, Candida albicans, Mycoplasma hominis*, and *Chlamydia trachomatis* [87]. Snake venom metallo-proteinases (SVMPs) are capable of bactericidal action by anionic site recognition and enzymatic degradation of phospholipid membranes. An SVMP from *Agkistrodon halys* inhibits the growth of Gram-positive bacteria like *Bacillus pseudomallei*, *Proteus vulgaris*, and *Staphylococcus aureus* [88]. Homodimeric phospholipase A2 is purified from *Bungarus faciatus* snake venom (BFPA); the cationic BFPA likely disrupts the anionic Gram-positive cell wall [89]. The antimicrobial activities of spider and scorpion venom peptides combined with commercial antibiotics have been evaluated; one peptide, Css54, works synergistically with rifampicin in killing rifampicin-resistant *Staphylococcus aureus* (55). Nanoparticles-venom toxin conjugates have anticancer and immune-modulatory activities; similar nanoparticle-venom conjugates could perhaps be employed as antibiotics. 

## 3. Novel Antimicrobial Molecules

**Figure 1:**
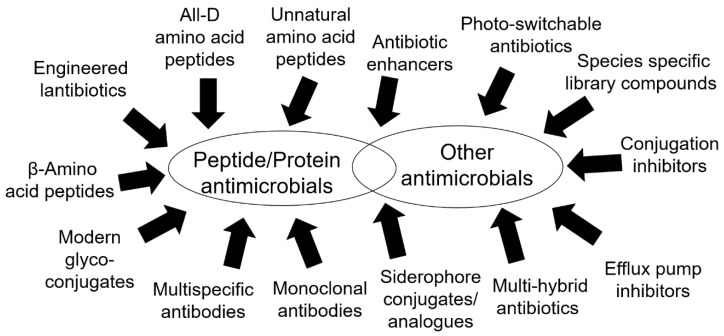
Major emerging classes of antimicrobial molecules. The division into peptides and non-peptides is not strict, since peptides can be antibiotic enhancers, siderophores, or parts of photo-switchable antibiotics.

Peptide chemistry and synthetic biology developed in a major way in recent years, expanding the scope for novel therapeutics. Antibodies are receiving more attention due to rising antibiotic resistance, while the development of inhibitors targeting efflux pumps, metal acquisition, and bacterial conjugation, as well as antibiotic enhancers and photo-activated molecules, has created other ways to tackle pathogens. Phage-based methods and breakthroughs, like CRISPR, may offer new routes to contain infections. Figure 1 summarizes some of the most interesting classes of novel antimicrobials that are emerging. 

### 3.1. Peptides and Peptide-Related Molecules

AMPs have served as antibiotics for decades now and new molecules continue to be developed. The majority of peptide antibiotics are cationic, because the outer bacterial cell membranes are typically rich in anionic lipids, unlike the zwitterionic eukaryotic cell membranes. Peptides encounter several obstacles in their early development, including proteolytic instability, short in vivo half-life, solubility issues, and poor bioavailability. Therefore, natural peptides are being used as scaffolds to design more effective therapeutics. Many broad-spectrum anti-biofilm peptides have been identified in recent years, since in vivo stability is less of an issue in wounds or epithelial surfaces [90]. The issues of bioavailability and in vivo stability continue to hamper the direct administration of many peptides, but their therapeutic properties could be improved by coupling to lipids, non-peptide antibiotics, photosensitizers, immunoglobulins, etc. [91].

Target selectivity is often an advantage of peptides over other antimicrobials. A 2-pyridone nonapeptide tested against *Neisseria gonorrheae* and *Neisseria meningitidis* in animal studies by PTC Therapeutics, USA, showed that only the *Neisseria* species were susceptible, leaving Gram-positive gut bacteria unaffected. Bactericidal action was suggested to occur via DNA-biosynthesis inhibition. A pH-active peptide has been developed against *Helicobacter pylori* [92]. This AMP adopts a random coil conformation at the physiological pH of the bloodstream or the intestines, while in the strongly acidic medium of the stomach it assumes a helical conformation containing positive charges, which destabilize the membranes of the pathogen. This strategy enables selective targeting of the pathogen, while leaving the larger gastrointestinal tract microbial community intact. 

Lipopeptides contain at least one lipid chain attached to the peptide head groups and are chiefly produced by bacteria to mediate competition with other bacteria, fungi, and viruses. They differ from other types of peptides due to the formation of self-assembled nanostructures contingent upon the intermolecular interactions between peptide molecules and the balance of hydrophilic and lipophilic groups. Modified derivatives of the antibiotic polymyxin containing substitutions to the cyclic heptapeptide and a fatty acid moiety were reported to be more potent than polymyxin itself by Cantab Anti-infectives, UK [93]. The lipopeptide Daptomycin (Cubicin™) [94] is used to treat systemic life-threatening Gram-positive bacterial infections, including multiple drug-resistant cases [95]. It alters the curvature of the bacterial cell membrane by aggregation, creating holes that leak ions. Rapid depolarization ensues, resulting in a loss of membrane potential leading to inhibition of protein, DNA, and RNA synthesis and killing the bacteria [96]. Surotomycin is a cyclic bactericidal peptide [97], which has been evaluated comparatively versus vancomycin and metronidazole for over 1000 intestinal strains [98]. It demonstrated lower recurrent rates and superior sustained clinical response rates for *Clostridium difficile* infections compared to vancomycin in phase 3 clinical trials [99]. 

Lantibiotics are peptides produced by Gram-positive bacteria containing rare post-translationally modified thioether amino acids, such as lanthionine (Lan) and methyllanthionine (MeLan); their biosynthesis, engineering, and mechanisms of action are reviewed elsewhere [100]. They are effective at low nanomolar concentrations, since Lan and MeLan induce the formation of intramolecular cyclic structures, offering resistance against bacterial proteases. Apart from the lanthionines, 17 other non-canonical amino acids, including didehydroalanine (Dha), didehydrobutyrine (Dhb), D-alanine, S-aminovinyl cysteine, lysinoalanine, and oxobutyrate, are found in lantibiotics. Due to their multiple modes of action, no notable resistance against lantibiotics has developed over decades. However, some bacterial strains are congenitally resistant against the prominent lantibiotic nisin [101]. Recently, two of the five lanthionine rings of nisin (rings D and E) were identified as playing a “fostering role” in the cleavage of nisin by the nisin resistance protein of *Streptococcus agalactiae*, paving the way for the synthesis of non-hydrolysable nisin analogues [102]. Bioengineering efforts have made it possible to modify the lantibiotic producing genes to create newer lantibiotic variants [103]. The LetM in vitro system was used to generate thioether and didehydro analogues of known non-lantibiotic peptides [104]. Pioneering in vivo studies enabled the introduction of dehydrated residues and thioether rings into non-lantibiotic peptides in *Lactococcus lactis* [105,106]. 

Glycoconjugate vaccines are carbohydrate antigens linked with proteins and were earlier created by randomly conjugating polysaccharides to basic or acidic residues on the surface of proteins. Mostly capsular polysaccharides were used, but glycoconjugation has been expanded to include O-antigens, exopolysaccharides, and teichoic acids [107]. Classical vaccines were obtained via isolation of polysaccharides directly from pathogens, but site-selective conjugations, protein modifications using unnatural amino acids and chemical or enzymatic modifications of native amino acids, and the bioengineering of carbohydrates (glycoengineering) are beginning to redefine this field [108]. Chemists can now also synthesize rare deoxy sugars found in bacterial cell surfaces, but not in humans, which may facilitate the development of new glycoconjugate vaccines [109]. 

All-D versions of peptides have been created by retroinversion (reversing the primary sequence and changing its amino acids to D-versions). However, this strategy is ineffective for helical peptides, since the direction of helix rotation does not change, leading to differences in orientation of particular groups with respect to the corresponding natural peptides. A new computational approach changes this situation by generating a D-version of every protein structure deposited in the PDB database [110]. After the D-PDB was thus created, all the D-protein α-helices were extracted, generating over 2.8 million files. Known protein-drug interactions were used to screen for D-helices that bound similarly to targets in silico to known protein and peptide drugs. Short D-strands created by retroinversion were used to link D-helices into D-analogues of known therapeutic peptides. This design strategy could enable the creation of novel completely synthetic AMP. 

A unique post-translational modification arising out of a previously unknown non-canonical protein-splicing reaction was shown to introduce β-amino acids into proteins [111]. The β-amino acids are structurally distinct from the α-amino acids that occur in natural peptides and proteins, in that they contain two carbons between the amino and carbonyl groups. The enzyme PlpXY catalyzes this reaction by removing tyramine from a tyrosine-glycine pair. It splices the gap by joining the orphan carbonyl group with the carbonyl group of the adjacent amino acid, thereby converting the adjacent amino acid into its α-keto-β-amino form. The β-peptides are resistant to proteases in the body, apart from providing an extra keto group as a chemical handle for further modification. Both these features make the PlpXY system an attractive means to potentially synthesize novel AMP. Recent years saw the expanding repertoire of non-canonical amino acids incorporated into proteins produced by ribosomes, which has been covered in detail elsewhere [112]. Rather than modifying t-RNAs, codons, and ribosomes, the number of bases usable by DNA to encode proteins has been augmented from four to six [113]. The two artificial bases, dNaM and dTPT3, undergo complementary base pairing not by hydrogen bonding, but by hydrophobic stacking. The expanded genetic code was inserted into *Escherichia coli*, whereby unnatural proteins could be produced via translation. This breakthrough in semi-synthetic genetic code expansion could soon enable the production of designer antibiotic peptides and new vaccines. 

### 3.2. Antibodies

The development of pathogen-specific monoclonal antibodies (mAb) is an emerging and highly promising area in infectious disease research. Passive immunization using serum therapy was used for the treatment of bacterial infections well before the discovery and development of antibiotics. However, due to the great success of antibiotics, the therapeutic potential of antibodies, the key molecular players of the adaptive immune system, has not been exploited fully. The successes of mAb-based therapies for cancers and autoimmune diseases and the increasing cases of antibiotic resistance have led many researchers to revisit this overlooked avenue of research. Currently, Palivizumab (brand name: Synagis), the only mAb-based antimicrobial on the market, is a humanized mAb approved against prophylaxis of respiratory syncytial viral (RSV) infection in neonates. Unlike antibiotics, mAb can be given prophylactically and have great scope in protecting vulnerable patients, such as those undergoing organ transplantations, bone-marrow transplantations, or in intensive care units. One of the key advantages of mAb over antibiotics is their exquisite target specificity and several are currently in the advanced developmental stages [114]. Thus, mAb could offer superior potency without adversely affecting the beneficial host microflora, while their high target specificity may provide less scope for selecting resistant strains. 

Unlike polyclonal antibodies, mAb are structurally uniform and well-defined, offering excellent sample-to-sample consistency and superior on-target activity. However, polyclonal antibodies can simultaneously target multiple epitopes by different isotypes, but mAb cannot. This limitation could be overcome by administering a cocktail of mAb, targeting not only different epitopes of the same antigen but different antigens. The target affinity of each individual mAb against its antigen epitope is higher than that of a polyclonal antibody and better protection from the pathogen would be accomplished. The production of human and humanized mAb has been achieved, whereby the risks of immunogenicity and toxicity are significantly lower than with conventional mAb. In a noteworthy example, Rossmann and co-workers developed a method for selecting B-cell producing antibodies with high opsonic killing against *Enterococcus faecalis* 12030 in healthy individuals, and produced fully human mAbs offering in vivo protection against many multi-resistant Gram-positive bacteria [115]. 

Existing diversity among bacterial strains and the rapid evolution of viruses that can modify their surface proteins may lead to weak binding of antibodies, and leading to failure to provide protection. However, antibodies can be altered with molecular biology methods, and these engineered antibodies could bind multiple targets rather than just one, thereby increasing their potency. Recently, an antibody that could bind three different targets on the HIV-1 envelope, namely the CD4 binding site, the V1V2 glycan site, and the MPER (membrane proximal external region) simultaneously, was reported [116]. These broadly neutralizing antibodies (bnAb) performed better than previously known bnAb against a mixture of simian-HIV strains in non-human primates. Multi-specific engineered antibodies could thus have wider applications in antimicrobial therapy. 

### 3.3. Phage-Based Strategies and CRISPR

Virulence reduction in bacteriophage resistant strains is known in multiple pathogenic bacteria [117]. It is a mechanism that bacteria cannot easily evade, since the same phages attenuate virulence both in natural environmental reservoirs and in humans. The ICP-1 phage of *Vibrio cholerae* targets the O1 surface antigen, which is important in human infections. *V. cholerae* usually evades this phage by mutations in the O1 antigen [118]. Similarly, the ICP-2 phage targets the outer membrane porin OmpU, which is also important in human infections. However, the ability to evade the ICP-1 and ICP-2 phages via mutations in the O1 antigen and the OmpU porin, respectively, compromises the ability of *V. cholerae* cells to survive the innate defenses of the human host. Similarly, 50–70% attenuation of virulence has been reported in phage-resistant *P. aeruginosa*, which harbor mutations in the O1 antigen and pilus [119]. Not all phage species are beneficial and they can incorporate genes for toxins and virulence factors into the genomes of bacteria. They can stay dormant for prolonged periods in bacterial cells, revive and infect other bacteria, spreading the virulence genes they carry in the process. Recently, a study of *Salmonella enterica* infections in mice reported that the host inflammatory response triggered the spreading of phage-based virulence among the bacteria [120]. Vaccination using engineered *Salmonella* that could not induce inflammation were shown to dramatically reduce the transmission of phage-based virulence between donor and acceptor strains, suggesting that suppressing host tissue inflammation is a way to contain such infections. Recent advances in the synthetic biology of phages make it possible to swap the tail domains of phages, thereby changing their host specificity [121]. Endolysins are enzymes secreted by bacteriophages and engineering them produces bactericidal “artilysins” [122]. These studies could stimulate the development of more generic phage-based approaches for controlling infections.

CRISPR (clustered regularly interspaced short palindromic repeats)/Cas9 was originally discovered in bacteria, where it is a form of adaptive immunity against bacteriophages [123]. RNA guides the Cas9 endonuclease to the phage DNA, which is cleaved and degraded. Following this discovery, other scientists repurposed CRISPR/Cas9 as a genome editing tool [124,125]. Soon, the self-targeting-induced cytotoxicity of CRISPR-Cas systems was reported, and researchers realized that the system could be used to immunize bacterial cells against plasmids containing multi-drug resistance genes [126,127,128]. CRISPR interference was shown to prevent the acquisition of virulence in vivo [129]. Cas9 was reprogrammed such that virulence genes could be selectively targeted with CRISPR-Cas9, enabling sequence specific killing of virulent strains, while leaving non-virulent ones of the same bacterium unaffected [129,130]. Soon afterwards, CRISPR-Cas systems were proposed as programmable antimicrobials; both endogenous and heterologous systems could selectively kill pathogenic bacteria, regardless of the target sequence [131]. While each engineered CRISPR-Cas system would be specific for one particular sequence, CRISPR itself could be broadly effective against any pathogenic bacterium. 

### 3.4. Miscellaneous Antimicrobials 

With increased understanding of the biology of specific diseases, new therapeutic enzymes, inhibitors, and vaccines specific to each pathogen may be produced. For example, the specific depolymerase EnvD [132], extracted from the soil bacterium, *Pusillimonas noertemannii* BS8, strips *Bacilllus anthracis* of its protective polypeptide capsule in the vegetative state, and enables mice to survive the inhalation of *B. anthracis* spores. Sugar-based bactericides have also been successful against *Bacillus anthracis* and *Bacillus cereus*, which often resist ciprofloxacin [133]. Species-specific compounds may be found in commercial compound libraries of potential antibiotics, even though they may have failed tests for broad-spectrum antibiotic activity. Therefore, repurposing old molecules from high throughput screening efforts may also be beneficial. Another strategy to limit microbial infection is to exploit the differences between the host and microbes in the structures of certain enzymes. Recently, a specific inhibitor, dubbed “Pseudouridimycin”, which binds to the active site of bacterial RNA polymerase, but not the human RNA polymerases, was reported [134]. Pseudouridimycin resembles the natural substrates of bacterial RNA polymerase and is expected to be effective against a range of drug resistant pathogens. 

The new field of photopharmocology involves the synthesis of “smart drugs”, whose activity can be controlled by light. The incorporation of “photo-switch” structures into therapeutic molecules allows for the remote-control of their properties, an approach well-suited to reduce the unintended environmental effects of antibiotics contributing to resistance. Photo-activated analogues of ciprofloxacin and gramicidin S were reported, with the former study describing a 50-fold increase in activity with light-activation [135,136]. Earlier studies were confined to molecules activated by UV light, which is toxic to living cells and does not penetrate tissue easily. However, recent breakthroughs enabled the use of visible light of different wavelengths for tuning the bioactivity of various photo-switchable trimethoprim analogues tested against *E. coli* [137]. 

Metal acquisition is crucial for pathogenic bacteria during infections, since the host imposes metal limitation as a form of “nutritional immunity” [138]. Bacteria sequester not only iron, but also manganese and zinc [139,140]. Synthetic siderophores are structural analogues of natural small molecules produced by bacteria mainly to chelate iron and used in quorum sensing, and are well-tolerated by animal hosts with MIC (minimum inhibitory concentration) values in the nanomolar range. Tethering synthetic siderophores to regular antibiotics could be a broad strategy against Gram-negative bacteria. An ampicillin-siderophore conjugate was shown to kill *P. aeruginosa*, while a cephalosporin-siderophore conjugate could kill *Acinetobacter baumannii* [141]. Due to their structural similarity with natural bacterial molecules, siderophore analogues are thought to evade the efflux pumps that render common antibiotics useless. 

In efforts to stem the rising tide of antibiotic resistance, small molecule inhibitors for multidrug efflux pumps and the bacterial conjugation machinery are being developed [142,143]. Efflux pump inhibitors (EPI) face the formidable obstacle that the multi-drug resistance (MDR) pumps of pathogens bind a large variety of chemically unrelated compounds. Nevertheless, efforts are on to identify the natural substrates of the MDR pumps, and a few generalizations could be made. Small polar compounds penetrated the MDR pumps easily, while zwitterions are also effective, since amphipathic cations are the assumed natural substrates. The inclusion of non-biological elements such as fluorine or boron increased the potency. A new, boron-containing class of antibiotic compounds with activity against Gram-negative bacteria was reported recently [144]. Bioassay-guided and high throughput strategies yielded EPI from plants and fungi, and synthetic analogues are known; EPI may perform better combined with other antimicrobials than alone [143,145]. 

Plasmid-borne resistance genes can be spread by bacterial conjugation, and the search for specific conjugation inhibitors (COINs) that block plasmid transfer is ongoing [142]. First, generation inhibitors were rather unspecific, having other confounding effects. The compound 2-hexadecynoic acid (2-HDA) is a simple compound readily accessible via chemical synthesis, which specifically inhibits conjugation in a broad range of donor cells [146]. Synthetic unsaturated fatty acid derivatives, in particular, are being used in efforts to prevent the transmission of antibiotic resistance among complex bacterial populations [147]. Targeting the bacterial assembly protein T4SS with small molecules was reported [148]. Other targets for COINs include the conjugative pilus and the relaxase protein, which initiates conjugation when the plasmid DNA is nicked. Luminescence-based high throughput conjugation assays are being applied to identify COINs in compound libraries [147]. 

Multi-hybrid antibiotics are large molecules containing structural domains from more than one antibiotic class. The best-known example is simocyclinone, which consists of distinct cyclic polyketide, deoxy sugar, polyene, and halogenated aminocoumarin moieties [149]. Using just 12 building blocks and one coupling reaction, about 75% of known polyene motifs could be synthesized [150]. Iterative biomimetic methods for polyketide synthesis have also emerged [151,152,153]. BGC-like modularity may soon be commonplace in natural product synthesis. Recent advances in synthetic chemistry have widened the range of sugar nucleotides and deoxy sugars accessible [154,155]. Together, these developments have created opportunities to create synthetic antibiotics in known classes as also synthetic and semi-synthetic multi-hybrid antibiotics by assembling separate domains. 

Simple molecules like bicarbonate (a widespread buffer in the human body) are being studied for modulating the effects of antibiotics. Bicarbonate decreases the pH gradient across the cell membranes of bacteria, enhancing the uptake of aminoglycosides, but suppresses that of tetracyclines [156]. The bicarbonate effect is specific to the mechanism of action of each antibiotic. In response to a drop in the pH caused by bicarbonate, cells transport more ions across the membrane. This increased charge distribution enables aminoglycosides to cross the membrane effectively, enhancing their effects. Bicarbonate also ramped up the effectiveness of the natural defense peptide indolicidin 100-fold in an assay against *E. coli* [156]. Combining the antibiotic minocycline with non-antibiotic drugs enhanced therapeutic efficacy, with some success against multi-drug resistant strains [157]. More widespread use of enhancers might extend the clinical lifetime of existing antibiotics. 

## 4. Antimicrobial Materials

Novel materials and technologies represent antibiotic-free ways to prevent microbial infections, but can also deliver antibiotics, as summarized in Table 2. 

### 4.1. Nanomaterials

The rapid growth of nanotechnology in the recent decades has opened up new possibilities for controlling microbial infection based on novel materials. Nanoparticles have been used in a variety of drug delivery systems in recent years. A recent study reported that silica nanoparticles containing peppermint oil and cinnamaldehyde could penetrate biofilms and kill drug-resistant bacteria [158]. A 99.9% kill rate with this method has been reported for the biofilm pathogens *P. aeruginosa, E. coli, Enterobacter cloacae*, and *S. aureus.* The acidic milieu of biofilms breaks down the silica of the nanoparticles, releasing the antibiotics within. This approach has been shown to work in cell cultures, and the main applications are expected to be disinfection and wound cleaning. Cinnamaldehyde triggers the activity of fibroblasts, which are immune cells involved in wound healing. This is significant since biofilms are often resistant to antibiotic penetration. 

Graphite, graphene, graphene oxide, and reduced graphene oxide were shown to have broad spectrum antimicrobial activities against bacteria and fungi [159]. Mixtures of graphene oxide with other simple substances have also been used for the same purpose. Graphene oxide kills bacteria by disrupting membranes as well as oxidation stress, whereas reduced graphene oxide traps bacteria [159]. The medical properties of silver and silver compounds have been known for 2000 years, but the antimicrobial activity of silver nanoparticles (size range 1–100 nm) has only been recognized since 2004 [160]. Silver nanoparticles can enhance the effect of commonly used antibiotics [161]. They have been used in wound dressings and commercial antimicrobial socks. Recently, silver nanoparticles were shown to kill the chlorine-resistant apicomplexan parasite *Cryptosporidium parvum* [162]. Many pathogens have become resistant to conventional disinfectants, necessitating increasing dosage and mixtures of disinfectants that may produce harmful by-products. Therefore, the incorporation of silver nanoparticles into filtration or water purification systems could prevent several types of water-borne illnesses. A novel example has been the use of multi-layered “Janus microbots”, containing silver nanoparticles as the disinfectant, a magnesium layer to help propel the microbots in water (via hydrogen evolution), and an iron layer to enable remote guidance via magnetism [163]. The microbots (along with the dead bacteria) can simply be collected after disinfection, thereby cleaning the water in one step and leaving no contaminants behind.

Nanoscale organic-inorganic hybrid particles, called “nanoflowers”, which are loaded with the enzymes, glucose oxidase (causing a pH change upon consuming glucose), and ConA (which binds the lipopolysaccharide O-antigen), have enabled the detection of food pathogens, including *E. coli* O157:H7, *Salmonella*, and *Listeria monocytogenes,* using simple pH meters [164]. While the application of modified viruses and different types of coated nanoparticles as antimicrobials has emerged in recent years, novel bacteriophage-shaped polymer nanoparticles have been shown to kill pathogenic bacteria, including some antibiotic resistant strains, such as multi-drug resistant *P. aeruginosa*, while leaving human cells unharmed [165]. Apparently, the particles’ geometry is the key property, enabling them to rupture the more curved bacterial cell membranes while not affecting the relatively flat lipid membranes of human cells. Other nanomaterials, such as chitosan-modified gold nanoparticles, can be conjugated with liposomes encapsulating antibiotics. Antibiotic release from liposome-conjugates triggered by bacterial toxins has been demonstrated [166]. When the conjugates encounter bacteria that secrete pore-forming toxins, the toxins insert into the liposomes, pores are formed, and the antibiotic within escapes. This mode of antibiotic release is more selective than unrestricted release from liposomes under physiological conditions. 

### 4.2. Materials and Techniques Targeting Biofilms

Biofilms contain a tough protective polysaccharide layer, conferring resistance to antibiotics. They form on the surface of medical implants and inside wounds. Recently several biomedical firms in Germany and the United States have been developing spider silk protein for coating catheter implants and medical devices. The build-up of bacteria on these device surfaces is prevented by spider silk, which could lead to significant savings for patients. Recombinant spider silk has been used in many studies and recent research also demonstrated the possibility of conjugating organic ligands to spider silk, creating variants with fluorescent or antibiotic properties using “click” chemistry [167]. It is possible that commercial antibiotic dressings made of spider silk will be available within the next few years, especially for treating conditions such as diabetic ulcers. 

Biofilm removal is a priority in the treatment of wounds, since the biofilm delays the healing process, resulting in a chronic wound infection. Electrical stimulation (ES) was originally used a century ago for wound treatment and has been applied in dermatology [168,169,170,171]. ES can eliminate biofilms from infected wound surfaces, enhancing wound healing. However, it was not widely applied because knowledge of the antibacterial mechanisms involved and standardized applications were lacking [168,169,170,171,172,173]. However, the rise in antibiotic resistance led renewed interest in ES. In a recent study, bacteria in biofilms were effectively killed by using electricity to release small amounts of hydrogen peroxide continuously via a conductive carbon fabric [174]. Electrochemical H_2_O_2_ generation thus represents a novel antibiotic-free way to keep wounds free from bacterial infection. Another strategy to tackle biofilms employs magnetotactic *Magnetospirillum* bacteria as swimmers delivering antibiotics into them [175]. The swimmers consist of mesoporous silica tubes filled with antibiotics, tethered to the magnetic bacteria. Under the influence of an external magnetic field, the swimmers enter the low oxygen environment of *E. coli* biofilms and the low pH of the biofilms triggered the breakdown of the silica microtubes, releasing the antibiotic. Although host immune reactions could destroy them, these hybrid swimmers could be explored further. 

## 5. Technological Advancements in Diagnostics and Screening 

Technologies for screening and diagnostics relevant to infectious diseases are exploding in the commercial sector. An overview of a few prominent examples is presented in Table 3. 

### 5.1. Infection Monitoring Technologies

Functional markers of antibiotic resistance that can be followed to identify pathogens are enzymes that directly degrade or modify antibiotics, mechanisms blocking antibiotics from reaching their targets, key resistance factors (specific genes and proteins), changes in intracellular composition or secreted products, and changes in cellular expression induced by antibiotics. Many newer methods offer results of antibiotic susceptibility testing in 2–6 hours instead of the days needed for traditional agar-based methods. Changes in morphology or other parameters can be monitored continuously in real time, an especially useful approach for well-known pathogens. With recent improvements to mass spectrometry techniques, such as MALDI-TOF MS and ESI-MS, and next-generation MS methods, new opportunities to track microbial diseases have arisen. MS-based strategies may involve tracking changes in metabolism through the use of stable isotope probes, such as amino acids. The detection of antibiotic response in real time, DNA sequencing based on MS technologies, the detection and quantification of metabolite profiles of growing cells, the direct detection of virulence factors, and following the penetration of antibiotics into cells have been facilitated by recent platforms. Other methods combine chromatography with mass spectrometry, such as GC-MS for metabolomics. One important development was the introduction of an MS-based spectral imaging system, which enables three-dimensional study of cellular contents at the nanoscale [176].

Monitoring the growth of pathogens in real time using microscopy has been important in identifying and treating infections. Manual monitoring is tricky and time consuming, and therefore, automated monitoring is growing. Automated digital microscopy in real time on agar media was introduced recently by Accelerate/QuantaMatrix, while real-time liquid phase microscopy in an ELISA layout was introduced by Biocell/Philips. Other than new-generation microscopes, devices such as a laser scatter device, which uses immobilized bacteria and detects morphological changes in single cells (BacterioScan Inc.), and a Fourier transform Infrared (FT-IR) spectrometer, which detects phenotypic changes upon interaction of cells and antibiotics in about 10 minutes (Spectromics Ltd, Manchester, UK), have entered the market. 

A microfluidics device that measures changes in electrical conductivity in droplets containing bacteria was able to determine if they were alive or dead [177]. This is interesting, since the method does not depend on cell type or concentration, and is “label-free”. Further development is expected to lead to array-format electrical sensors for lab-on-a chip applications. Microfluidics has also been used in combination with acoustic tweezers to sort cells according to their response to drug candidates [178]. Another recent development is a chip which enables co-cultivation of gut microbes and epithelial cells of the human intestines [179]. Such “Gut-on-a-chip” technologies help simulate the human gut in health and disease, contributing to better understanding and future therapeutic interventions. Microfluidics for monitoring antibiotic resistance is also a rapidly advancing field [180]. Microfluidic chips for single cell sequencing of uncultured microbes have emerged, which makes high-throughput culturing and genome sequencing of unknown microbes possible [181]. 

The infection of implants is a major issue affecting the outcome of surgery, especially for orthopedic or trauma patients. Implants may also loosen aseptically due to mechanical causes or due to septic infections, but traditional radiology and biopsy culture-based methods cannot distinguish between the two scenarios. An AMP called UBI was tethered to a hybrid label containing both a fluorescent group and a radioactive isotope, and it was demonstrated that the AMP-conjugate selectively accumulated at the site of bacterial infections [182]. Distinguishing septic infection from inflammation has thus been enabled by a specific labelling approach. 

### 5.2. Direct in-Sample Technologies

The need for enrichment, culture, or sample preparation is a major reason for the delay between obtaining clinical samples and analyzing them. Therefore, directly in-sample detection methods for pathogens were launched. In a magnetic resonance-based platform introduced by T2 MR, proprietary particles are coated with specific binding agents. Encountering a target, the particles bind to and cluster around it, modifying the arrangement of water molecules in that sample and thereby changing the magnetic resonance signal. The change in the signal correlates with the absence, presence, or concentration of the target, tolerating interferences likely present in bodily fluids. Testing for amplified DNA probes attached to particles using magnetic resonance spectra combined with PCR; this system can deliver results in about 2 hours. The ability to analyze antibiotic susceptibility or identify multi-drug resistance without further processing of clinical samples is advantageous. Geneweave/Roche launched the “Smarticles” technology based on the delivery of “smart particles” with specific probes. Upon binding of targets with the probes, these “Smarticles” generate luciferase-mediated fluorescence in live cells directly in clinical samples, enabling rapid automated analysis. Lifescale Analytics introduced a device which measures changes in cell mass directly on clinical specimens. The Oxford Nanopore MinION sequencer offers portability, speed, and relatively long read lengths. For example, MinION-based analysis of urine samples generated data enabling the direct detection of pathogens four times faster than traditional culture-based methods [183].

### 5.3. Point-of-Care Devices

The development of integrated diagnostic work-flows designed for the Point-of-Care (the doctor’s office or health center), rather than specialized medical labs, promises to reduce the delay between sample collection and analysis. Speeding up diagnosis could help the patient before the disease proliferates, an important consideration for tackling fast-progressing infections, epidemics, or infections in remote or low-income settings with unreliable power and transport facilities. Recently, the fabrication of a microfluidic device able to separate serum from cells in a single drop of blood was reported [184]. PCR amplification of DNA from the samples could be performed in situ, since the necessary reagents were patterned into wells present in the device. Heating the whole assembly in an oven enabled the detection of viral RNA or bacterial DNA in the range of 10^1^–10^5^ copies/µL. A rapid and inexpensive assay to distinguish between influenza and bacterial infections has been recently developed [185]. The test uses an artificial sialic acid conjugate of galactose that is cleaved only by viral, but not bacterial, neuraminidases, and the released galactose can be detected by a common hand-held glucose meter. The integration of new biochemical tests with existing medical devices and emerging Point-of-Care devices could potentially revolutionize the field of infection diagnostics. 

## 6. Ecological Management 

It has become clear in recent years that the resident microbiome is critical to human health. Rather than attacking pathogens directly with drugs, ameliorating the damage to the host via microbiome engineering might be a preferable strategy for countering infectious diseases. This could reduce the need for antibiotics as a broad catch-all strategy for countering microbial disease. In order to design future interventions into the microbiome, ecological interactions between members of microbiomes and host-microbiome interactions need to be studied and understood. 

### 6.1. Probiotics, Synbiotics and Prebiotics

Two approaches involving microbiome-related interventions are worth mentioning, namely probiotics and prebiotics. The Nobel laureate Élie Metchnikoff had in 1907 already suggested that "the dependence of the intestinal microbes on the food makes it possible to adopt measures to modify the flora in our bodies and to replace the harmful microbes by useful microbes” [186]. Currently, “probiotic” is the designation for microorganisms, which upon ingestion are beneficial for humans or domestic animals [187,188]. Several reviews have been written on the subject and many commercial food products and supplements are being sold as purported “probiotics”. The benefits of *Lactobacillus* and *Bifidobacterium* species have been researched intensively. Lesser known strains include *Bacillus clausii* Strain O/C, which secretes factors inhibiting the cytotoxic effects of the toxins from *Clostridium difficile* and *Bacillus cereus* [189]. *Clostridium tyrobutyricum* was shown to be protective against induced ulcerative colitis in mice [190]. 

A prebiotic is defined as a “selectively fermented ingredient that allows specific changes, both in the composition and activity in the gastrointestinal microflora that confers benefits upon the host’s well-being and health” [191,192]. Synergistic combinations of probiotics and prebiotics are called synbiotics [191]. Prebiotics commonly exhibit the properties of resistance to gastric acid, hydrolytic mammalian enzymes, and absorption in the gastrointestinal tract, as well as fermentation by the microbes of the intestine, and specific stimulation of bacterial activity and growth in the intestines, leading to the maintenance of host health and well-being [192,193]. Presently, a few categories of compounds considered to satisfy the above definition of prebiotics include fructo- and galacto-oligosaccharides, lactulose, and indigestible carbohydrates. The indigestible carbohydrates include inulin, pectins, gums, cellulose, and hemicellulose among the larger polysaccharides, and oligosaccharides, sugars, and alcohols, which cannot be absorbed by the host intestines. Fructo-oligosaccharides and inulin are hydrolyzed by *Bifidobacteria* and stimulate their growth, which in turn contributes to intestinal homeostasis and inhibits the growth of pathogens [194,195,196].

Short chain fatty acids (SCFA), for example, acetic acid, propionic acid, and butyric acid, are produced as the end products of carbohydrate and amino acid catabolism. Epithelial cells in the colon derive energy from gut microbes performing carbohydrate fermentation [197]. SCFA are able to induce apoptosis (programmed cell death), lowering the risk of developing not only gastrointestinal disorders, but also cardiovascular diseases and cancers [198,199]. Butyrate production by resident Clostridium species in the gut induces colonic regulatory T-cells in the host, suppressing its immune reactions, including the inflammation of the intestinal mucosa, which is implicated in many diseases [200]. More research into simple small molecules able to modulate the microbiome, whether or not strictly prebiotic, may prove fruitful. 

### 6.2. Understanding Ecological Interactions in Health and Disease

#### 6.2.1. Host-Microbiome Interactions

Members of the resident microbiome can switch their lifestyle, from beneficial or just commensal to pathogenic, sometimes triggered through relocation of a given microbial species from its normal niche to another where it is normally absent. Injury or trauma, stress, or reduced immunity of the host can also trigger the switch. *Bacteroides thetaiotaomicron* is a beneficial member of the human gut microbiome, which ferments carbohydrates and stimulates host immune development. However, migration to other organs, such as the brain, lungs, or liver, causes it to become pathogenic [201]. *Streptococcus pneumoniae* is a commensal of the human respiratory system, where it produces hydrogen peroxide to kill competing bacteria. In the bloodstream, lungs, and inner ear, it causes infection by secreting a pore forming toxin [202]. *S. aureus* is a normal resident of human skin, and nasal cavity which can cause systemic sepsis if it enters the bloodstream [203]. 

The interplay between human hosts and bacterial pathogens is well-studied. However, in many cases, an important role could be played by the bacterial community in the development of infectious disease. Antibiotic treatments often induce diarrhea due to the disturbances to the indigenous microflora of the gut. The major mechanism is proposed to be the alteration of intestinal barrier function and reduced resistance to pathogens and altered metabolism of SCFAs and bile acids [204]. Probiotics, including the yeast *Saccharomyces boulardii* and many *Lactobacilli*, such as *Lactobacillus acidophilus, Lactobacillus rhamnosus* GG, *Lactobacillus delbruckii*, and *Lactobacillus fermentum,* effectively reduce the occurrence of antibiotic-induced diarrhea [205]. Of particular concern is the increased vulnerability of patients to *Clostridium difficile* infections followed prolonged antibiotic administration [206]. Eradication of other members of the gut microbiome, which are normally protective, may enable *Clostridium difficile* to thrive. This “niche vacation” hypothesis is further supported by the fact that *Clostridium difficile* infections were ameliorated with species present in the fecal pellets from healthy volunteers used in “faucal transplant therapy” [207,208]. The material used for the therapy later became available in capsule form [209]. Prior to bloodstream infection by vancomycin-resistant *Enterococcus* (VRE) species in immuno-compromised patients, *Lactobacilli* and related genera in the gut are replaced by *Clostridia* and *Enterobacteriaceae* [210]. Not surprisingly, some VRE patients respond well to fecal transplants, with the beneficial microbe being identified as a *Barnesiella* species [211]. Even two different strains of the same species can interact; horizontal gene transfers conferring plasmid-borne resistance to multiple antibiotics were already reported by 1974 [212]. However, in the context of the human microbiome, antibiotic-sensitive commensal bacteria can also compete out their resistant peers of the same species, as reported for *Enterobacter faecium* [213]. Interestingly it was reported that commensal *Entercoccus faecalis* strains turned the mobile genetic elements of the vancomycin-resistant *Enterococcus faecalis* V583 strain against the latter, effectively killing them [214].

In the ecological sense, infection can happen when new niches are opened up due to direct damage to the host itself, or when existing niches are emptied of beneficial bacteria, creating a colonization opportunity for pathogens. “Colonization resistance” is the protective effect of the microbial community composition against pathogens [215]. Direct antagonism between two species was reported in a study of mice; the *Escherichia coli* strain Nissle 1917 could prevent *Salmonella typhimurium* infection by sequestering iron [216]. *Vibrio cholerae* contains several genes enabling it to resist the Type VI secretion systems of many normal intestinal microbes [217]. Several members of the microbiota are capable of inhibiting colonization by *Vibrio cholerae*, and recovery from cholera involves recolonization by indigenous “healthy bacteria” [61]. Subjects with a certain skin microbiome composition were resistant to a sexually transmitted disease, and knowing the skin microbiome composition could be a strategy to identify people at risk of infection [218]. There are differences between single and mixed infections, due to cooperative and competitive interactions between bacteria involved in quorum sensing [219]. In synthetic media, *Pseudomonas aeruginosa* cells with mutations in the *lasR* gene are out-competed by the wild type, since the former do not respond to quorum sensing. However, in mixed culture, *lasR* mutants of *Pseudomonas aeruginosa* become “social cheaters”, gaining an advantage by exploiting the exoprotease production of wild-type cells [219,220]. This could explain the observation that *lasR* and other quorum sensing mutants persist in infections, even though “loss of function” mutations may be deleterious. 

#### 6.2.2. Inter-Microbial Interactions

Bacteria and fungi form many kinds of extracellular and intracellular associations. The gene expression of bacterial as well as fungal pathogens can change in the presence of the other. Bacteria-fungi interactions (BFI) are known in cheese, where the hyphae of Mucor fungi form “superhighways”, through which the bacterial pathogens, such as *Listeria*, can spread [221]. There are many bacterial-fungal co-infections of burn wounds and keratitis [222,223,224,225,226,227,228,229]. The lungs of immuno-compromised individuals are often the sites of bacterial-fungal “superinfections”, notably involving *Pseudomonas aeruginosa* and fungi, such as *Candida albicans* and *Aspergillus fumigatus*, which may worsen lung function [227,230]. Clinical studies have been focused on the yeast *Candida albicans* occurring in the buccal, dermal, and intestinal microbiomes, and a causative agent for a number of infections [231]. In the presence of *Candida albicans, Staphylococcus aureus* shows enhanced biofilm formation and resistance to vancomycin in serum [232,233,234]. *Escherichia coli* and *Staphylococcus aureus* are synergistic *Candida albicans* co-pathogens and cause increased mortality in animal models of polymicrobial peritonitis [235,236,237]. Bacterial small molecules affect the critical yeast-hyphal morphological transition of fungi during pathogenesis. This transition in *Candida albicans* is inhibited by SCFA from lactic acid bacteria [238] and mutanobactin A from *Streptococcus mutans* [239]. Hyphal growth is induced by cell wall peptidoglycan, while *Candida albicans* farnesol production modifies bacterial virulence by interfering with quorum sensing [240,241]. *Cryptococcus neoformans* utilizes homogentisic acid produced by *Klebsiella aerogenes*, as a precursor for melanin pigments, which protect against ultraviolet radiation and cause increased virulence [242,243]. 

The presence of antibiotics exerts a selective pressure, which favors the survival of antibiotic resistant fungi and bacteria. Physical contact and environmental cues, such as nutritional conditions, can modulate co-adherence between *Candida albicans* and oral bacteria [244]. *Staphylococcus aureus* associates with the hyphae of *Candida* species, which provides a mechanism for the bacterial invasion of otherwise inaccessible tissues, such as epithelial layers [245]. *Candida albicans* strongly enhanced the biofilm formation on polystyrene by *Staphylococcus aureus*, with the latter adhering to the fungal hyphae in a poly-microbial biofilm rather than the plastic substrate [233]. Similar effects may be important for the colonization of medical devices by a range of microbes [246]. Mixed bacterial-fungal biofilms are of considerable medical interest due to their prevalence in certain infections [247] and on the surface of medical devices, such as catheters, prostheses, and mechanical ventilators [248,249]. However, clinical therapies have been developed to target either fungal or bacterial infections, failing to consider co-infection, with the mixed communities showing virulence and resistance properties significantly different from those of the single species [228,229]. An understanding of BFI in human health is, therefore, an important emerging medical challenge. However, not all BFI are pathogenic to animal hosts. The first probiotic BFI effect was demonstrated by the use of *Saccharomyces boulardii* to combat antibiotic-associated diarrhea caused by the loss of indigenous microbes after antibiotic therapy and subsequent colonization by bacterial pathogens like *Clostridium difficile* [250]. The protective effect of *Saccharomyes boulardii* has been attributed to mechanisms, such as the degradation of *Clostridium difficile* toxins by yeast proteases and the alteration of *E. coli* lipopolysaccharide by a yeast phosphatase [251,252,253]. In the presence of *Saccharomyces boulardii*, reductions in bacterial adherence, colonization of intestinal epithelial cells, and expression of virulence factors were reported for *Citrobacter rodentium* colon infections [254]. 

The role of predators in the ecology of macroscopic animals is well-established. Predation probably plays an equally important role in microbial communities, but has received much less attention. Obligate predatory bacteria that consume Gram-negative bacteria for nutrients and reproduction, namely the *Bdellovibrio* and like organisms (BALOs), which include the genera *Bdellovibrio*, *Bacteriovorax*, *Peredibacter*, and *Halobacteriovorax* [255,256]. BALOs were first used in agriculture in the 1970s as a form of biological control for *Pseudomonas syringae* bacterial blight disease in soybeans [257]. Studies on *Bdellovibrio* and *Halobacteriovorax* species increased in recent years and predatory bacteria are beginning to be recognized as keystone species in their ecosystems, termed “living antibiotics” [258]. Both *Bdellovibrio* and *Halobacteriovorax* species can attack a variety of Gram-negative bacteria, such as *Caulobacter crescentus* [255], *Escherichia coli*, *Klebsiella pneumoniae* [259], and *Salmonella* species [259], and are therefore important in controlling potential human pathogens in soil and aquatic reservoirs. Control of *Vibrio cholerae* in white-leg shrimps using *Bdellovibrio bacteriovorus* was demonstrated in China recently [260]. The use of predatory bacteria to check the proliferation of pathogens in aquaculture and animal husbandry, where antibiotic resistance is already a major issue, could be crucial in controlling future outbreaks of food-borne diseases.

### 6.3. Wider Ecology and One Health

It is known that many pathogens have distinct life-cycles or life-cycle stages in different species of hosts, some of which are merely reservoirs, while others suffer from disease symptoms. *Salmonella enterica*, a human pathogen, benefits from interactions with plant pathogens [261]. It enters soft rot lesions in tomatoes and thrives in the company of *Pectobacterium carotovorum*, which secretes enzymes to degrade plant cell-walls that it lacks. Other bacteria also protect plant surfaces, such as edible leaves, against colonization by human pathogens such as *Eshcerichia coli* O157:H7 [262]. Some human pathogens have reservoirs in non-human animals and some have many hosts; for example, *Salmonella enterica* serovar Typhimurium can infect around 40 metazoan species including humans [263]. Therefore, the divide between human and veterinary medicine could be affecting efforts to curb infectious diseases.

In the One Health view, human beings, farm animals, crops, and wildlife form an inter-connected system. Managing disease outbreaks or the flow and disposal of antibiotics into ecological reservoirs, such as soil or water, should not be isolated exercises aimed solely at human beings. Widespread antibiotic resistance occurs among farm animals and in food products derived from them [264]. Pets, feral animals, and wildlife also show the same trend [265]. In this context, an integrated approach to monitor and restrict the use and dispersal of antibiotics via animal husbandry, medical waste, and wastewater would certainly be beneficial in the long term. It has been suggested that the bush-meat trade in infected bats reaching new population centers at the edge of formerly undisturbed forests could have played a role in the most recent Ebola outbreak in West Africa. Thus, the opening up of forests due to human population pressures and climate change may bring diseases once confined to small populations of forest-dwellers into more thickly populated urban areas in the economically less-developed regions of the world. This calls for the inclusion of animal and environmental health in public health programs designed for poorer countries. Richer temperate zone countries in the could also be affected by an increased incidence of infectious diseases spreading farther due to global warming enhancing the survival rates of insects and other arthropod vectors at higher latitudes. Thus, climate change would need to be taken into account in public health plans for these countries.

## 7. Conclusions

There have been a number of promising developments in the field of antimicrobials recently, as outlined in this review. It takes 10–15 years for a new drug molecule to reach the market from its initial discovery. Most of the molecules discussed in this article are still in the early stages of discovery; a few have made it to clinical studies and they are listed in Table 4. Those in phase 3 clinical studies could be expected to be on the market in the next few years. Nevertheless, many challenges remain and technical developments alone will not be enough; major policy decisions and practical measures are needed to fight infectious diseases. Even so, developments in such areas remain mixed. For example, several large food corporations promised action on checking misuse of antibiotics in animal husbandry, but troubling questions remain on the implementation, due to a lack of binding rules. Also, antibiotic production facilities in many countries are not equipped to deal with pharmaceutical waste, and as a result, widespread antibiotic resistance has been reported among bacteria in wastewater and soil adjacent to effluent treatment plants [266,267]. The withholding of negative data from clinical trials and the suppression of the structural details of new drugs via commercial patents also hinder the multinational, multi-institutional, and multidisciplinary approaches needed to tackle epidemic situations, like the 2015 Ebola outbreak. However, the development of the Zika vaccine demonstrated more concerted action in the Americas, which is encouraging. 

Up to 80 percent of antibiotics are prescribed by healthcare providers or purchased directly by consumers or caregivers, without prescription in some countries [268]. Non-prescription antibiotics were more likely to be used by patients in public health facilities, who were often less educated and had lower annual incomes [269]. Greater efforts are thus needed in raising awareness about antibiotic misuse among sections of the public with lower education and socio-economic status. Careful drug management can reduce the incidence of drug-resistant infections and prolong the effectiveness of antibiotic arsenals, according to a new study of ciprofloxacin use [269]. Under a stewardship program, whereby a multidrug panel was tested with the mandate that ciprofloxacin susceptibility be reported if, and only if, there was no other susceptibility. A strong reduction in ciprofloxacin use was observed, which correlated with increasing susceptibility in clinical *Eshcerichia coli* isolates towards this antibiotic. Some countries already have stewardship programs in place, and a wider adoption of such programs at the national and regional level could be beneficial globally in limiting the rise of resistance towards various antibiotics. In the last few years, governmental agencies in the EU and the US have been more proactive in framing guidelines for antibiotic use, which is encouraging. More coordination between different players in the public health scenario and concerted efforts are needed to effectively tackle the growing threat of antibiotic resistant infections.

## Figures and Tables

**Table 1:** Emerging targets for antibiotic research and development, current discovery approaches, and potential sources for novel antibiotics.

Novel Targets Identified with References	Discovery Approaches	Sources
Essential amino acid biosynthesis [7,8,9,10,11,12,13,14]	Informatics-based mining	Medicinal plants
Cell wall lipid biosynthesis [15,16]	Cryptic Biosynthetic Gene Clusters (BGC) activation Antibiotic stimulation Quorum sensing signals Specific pathway inhibitors	Marine invertebrates
Lipid insertion enzymes [17]		Insect and vertebrate symbionts
Metal chelator biosynthesis [18]		Microbial co-cultures
Quorum sensing metabolism [19,20,21,22,23,24,25,26,27,28,29,30,31]	BGC engineering Special plasmids Engineered pathways Replacing native promoters	Endophytic fungi and bacteria
Clp proteases [32,33]		Uncultured microbes
Cyclic-di- Guanosine monophosphate (GMP) levels [34]		Skin, blood, venoms

**Table 2:** Current antimicrobial materials and technologies for infectious disease prevention, in preclinical development, and as vehicles for antibiotic delivery.

Prevention	Preclinical	Antibiotic Delivery
Graphene and silver-based nanomaterials Organic-inorganic hybrid nanoparticles Microbots for water treatment	Phage-patterned nanoparticles	Silica nanoparticles Nanoparticle-liposome conjugates
Engineered spider silk	Electrochemical H_2_O_2_ generation	Hybrid bacteria-nanoparticle swimmers

**Table 3:** Major new technologies in infectious disease diagnostics.

Monitoring Technology Types	Direct in-Sample Methods	Point-of-Care Devices
Mass spectrometry-based	Magnetic resonance-based	Microfluidic blood serum separator
Automated imaging	Smarticles	Portable influenza tester
Microfluidics-based	DNA sequencing-based	-
Label-based	-	-

**Table 4:** Some antibiotics recently evaluated in clinical studies.

Drug Molecule with Reference	Clinical Phase	Medical Condition
POL70780/Murepavadin [270]	Phase 3	Pneumonia
Surotomycin [271]	Phase 3	Diarrhea
Cethromycin (semi-synthetic) [272]	Phase 3	Pneumonia
Solithromycin (semi-synthetic) [273,274]	Phase 2	Uncomplicated urogenital gonorrhea, Pneumonia
